# PLS Subspace-Based Calibration Transfer for Near-Infrared Spectroscopy Quantitative Analysis

**DOI:** 10.3390/molecules24071289

**Published:** 2019-04-02

**Authors:** Yuhui Zhao, Jinlong Yu, Peng Shan, Ziheng Zhao, Xueying Jiang, Shuli Gao

**Affiliations:** 1 School of Computer Science and Engineering, Northeastern University, Shenyang 110819, China; jianren_d@163.com (J.Y.); 13081850350@163.com (Z.Z.); xueying.jiang@163.com (X.J.); 15238247216@163.com (S.G.); 2 College of Information Science and Engineering, Northeastern University, Shenyang 110819, China; peng.shan@neuq.edu.cn

**Keywords:** calibration transfer, NIR spectroscopy, PLS, quantitative analysis model

## Abstract

In order to enable the calibration model to be effectively transferred among multiple instruments and correct the differences between the spectra measured by different instruments, a new feature transfer model based on partial least squares regression (PLS) subspace (PLSCT) is proposed in this paper. Firstly, the PLS model of the master instrument is built, meanwhile a PLS subspace is constructed by the feature vectors. Then the master spectra and the slave spectra are projected into the PLS subspace, and the features of the spectra are also extracted at the same time. In the subspace, the pseudo predicted feature of the slave spectra is transferred by the ordinary least squares method so that it matches the predicted feature of the master spectra. Finally, a feature transfer relationship model is constructed through the feature transfer of the PLS subspace. This PLS-based subspace transfer provides an efficient method for performing calibration transfer with only a small number of standard samples. The performance of the PLSCT was compared and assessed with slope and bias correction (SBC), piecewise direct standardization (PDS), calibration transfer method based on canonical correlation analysis (CCACT), generalized least squares (GLSW), multiplicative signal correction (MSC) methods in three real datasets, statistically tested by the Wilcoxon signed rank test. The obtained experimental results indicate that PLSCT method based on the PLS subspace is more stable and can acquire more accurate prediction results.

## 1. Introduction

In the past few decades, near-infrared spectroscopy (NIR) has been widely used in various fields, because of its fast speed and the fact that it does not cause damage to sample characteristics. These areas include pharmaceutical [1,2,3], biomedical [4], petrochemical [5], agricultural [6,7], food [8,9,10]. In the NIR analysis, the most frequently used multivariate calibration techniques are partial least squares regression (PLS) [11,12] and principal component regression (PCR) [13,14]. However, the established calibration model is often outdated or unsuitable for new samples due to factors of the diversity of measuring instruments and measuring environments, as well as the variability of the materials being measured. New samples refer to any samples not included in the calibration model, such as those samples collected at different times or with different instruments. Frequent calibration is not desirable because a large amount of time and resources are devoted to establishing calibration models. One advisable option would be to carry out the calibration transfer.

Numerous relevant calibration transfer methods have been proposed in articles. In general, these methods can be divided into two types: transfer standard and non-standard. The transfer standard requires the same standard samples to be measured on the master instrument and the slave instrument. In this type of method, according to the stages in which the adjustment occurs are further divided into four types.

The first type is the method of correcting the slave spectra. In the standard samples, the slave spectra are made as close as possible to the corresponding master spectra by a transfer matrix. The most widely used are direct standardization (DS) and piecewise direct standardization (PDS) methods [15,16]. In the PDS method, the transfer relationship between the master spectra and the slave spectra from the sliding window is established at each wavelength of the master spectra, and finally a band-shaped transfer matrix is formed for correcting the slave spectra.

The second type is the method of simultaneously correcting the master spectra and the slave spectra. Commonly used is calibration transfer by the generalized least squares (GLSW) method [17,18]. GLSW uses the difference between the standard set of the master instrument and the slave instrument to build the weight matrix, and then uses the weight matrix to reduce the weight of spectral feature to be suppressed. A detailed description of the weight matrix is provided in [17] and [18].

The third type is the method of correcting the predicted values. Mainly the slope and bias correction (SBC) method [19], this method considers that there is a linear relationship between the predicted values of the slave spectra obtained by the master spectral model and the response variable, usually using ordinary least squares method to calculate this relationship. The predicted values are then corrected using this relationship.

The fourth type is the projection method. For example, calibration transfer method based on canonical correlation analysis (CCACT) [20], which uses CCA to find the set of canonical variables that are maximally correlated between the standard set of the master instrument and the slave instruments. Further explore the transfer relationship between the two canonical variables.

In practical applications, it is difficult or even impossible to measure the same samples on two instruments due to the position of the measuring instrument and the stability of the samples, etc. At this time, it is necessary to use a method that does not require measurement of the same standard samples, that is, a non-standard method. These methods are mainly divided into two types.

One is the signal preprocessing method, which removes the baseline offset and the linearly sloped baselines by simple mathematical operations of the first derivative and the second derivative. Common methods include multiplicative signal correction (MSC) [21], finite impulse response (FIR) filtering [22], generalized moving window MSC (W-MSC) [21], OSC [23,24], etc., wherein FIR and MW-MSC are variants of MSC. However, it must be noted that these simple preprocessing methods do not handle complex changes between the master spectra and the slave spectra.

The other is the projection method. It includes transfer component analysis (TCA) [25] and kernel principal component analysis (KPCA) [26]. TCA projects the master spectra and the slave spectra into a common feature space in which the distribution of the master spectra and the slave spectra are as similar as possible while retaining the key properties of the spectra. TCA and KPCA use different kernels, so they can cope with nonlinear and more complex changes in the spectra.

In this paper, a novel projection method is proposed, which is a feature transfer model based on PLS subspace (PLSCT). PLSCT establishes the PLS model of the calibration set of the master instrument firstly, constructing a low-dimensional PLS subspace, which is a feature space constructed by the spectral feature vectors. The PLS model is then used to extract the predicted features of the master spectra and the pseudo predicted features of the slave spectra, that is, to project all spectra of the master instrument and slave instrument into this PLS subspace. Then, the ordinary least squares method is used to explore the relationship between the two features in the identical PLS subspace, the relationship will then be resorted to construct a feature transfer relationship model.

Notice that the pseudo predicted feature of the slave spectra is acquired by the PLS model established by the master instrument rather than the PLS model of the slave instrument. And PLSCT does not need the response variable corresponding to the standard set. In addition, compared with PDS, PLSCT corrects the feature of the spectra rather than the spectra. In contrast to CCACT, PLSCT uses PLS to find the covariance between the spectra and the response variable, instead of using CCA to find the correlation between the master spectra and the slave spectra.

In order to validate the performance of the PLSCT model, we not only compare its prediction results against those of the SBC, PDS, CCACT, GLSW, and MSC methods, but also apply the Wilcoxon signed rank test [27] to determine whether PLSCT is statistically significantly superior to other models. The experiment was conducted in three real near-infrared datasets. By analyzing all the experimental results, we conclude that the PLSCT can significantly reduce the prediction error.

## 2. Results and Discussion

### 2.1. The Analysis of the Corn Dataset

First of all, Table 1 lists the latent variables (LVs) and the root mean square error of prediction (RMSEP) of Calibration, Direct transfer and Recalibration. The RMSEP was 0.010156 when using the calibration model of the master instrument to predict the spectra of the test set measured on the master instrument. However, when directly using the calibration model of the master instrument to predict the spectra of the test set measured on the slave instrument, the RMSEP was 1.41931, which indicates that if the model of the master instrument is directly applied to the slave instrument, a large prediction error will be generated.

**Figure 1:**
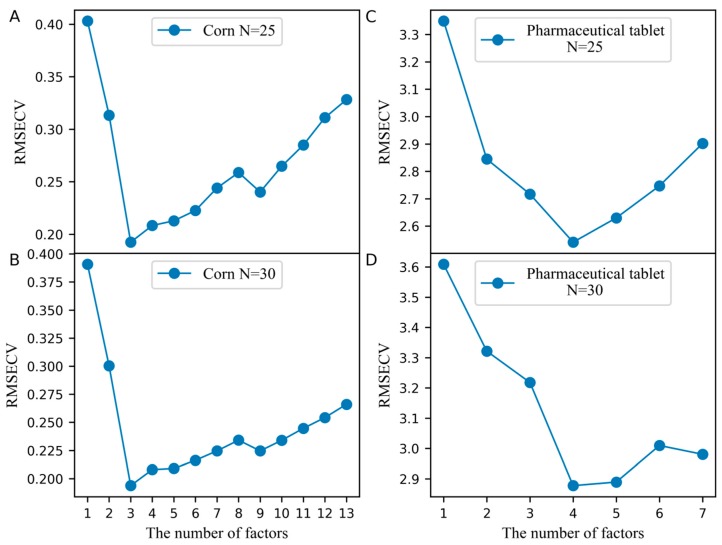
The effect of selecting the number of factors when building ${\tilde{T}}_{\mathrm{std}}^{s\_m}$ and ${\hat{T}}_{\mathrm{std}}^{m}$ on the cross-validation error. (**A**) Corn dataset and the number of the samples in the standard set is 25, (**B**) Corn dataset and the number of the samples in the standard set is 30, (**C**) Pharmaceutical tablet dataset and the number of the samples in the standard set is 25, (**D**) Pharmaceutical tablet dataset and the number of the samples in the standard set is 30.

The number of the factors for constructing the pseudo predicted feature matrix from the standard set of the slave spectra (${\tilde{T}}_{\mathrm{std}}^{s\_m}$) and the predicted feature matrix from the standard set of the master spectra (${\hat{T}}_{\mathrm{std}}^{m}$), which is a key parameter in the PLSCT model, was determined by leave-one-out cross-validation. Figure 1A,B illustrates the effects of selecting the number of factors used to build ${\tilde{T}}_{\mathrm{std}}^{s\_m}$ and ${\hat{T}}_{\mathrm{std}}^{m}$ on the cross-validation error when the number of the samples in the standard set is set to 25 and 30. From the results in Figure 1A,B, inferring that when the number of the samples in the standard set is set to 25 and 30, the number of factors should be set to 3. At this time, the root mean square error of cross-validation (RMSECV) reached the minimum and PLSCT achieves the best performance.

**Figure 2:**
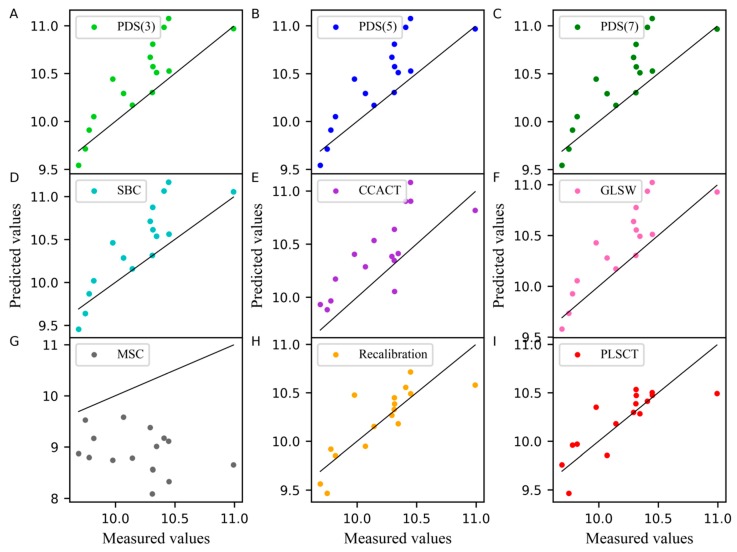
Measured values versus predicted values of water content for corn dataset as determined by (**A**) piecewise direct standardization with a window size of 3 (PDS(3)), (**B**) piecewise direct standardization with a window size of 5 (PDS(5)), (**C**) piecewise direct standardization with a window size of 7 (PDS(7)), (**D**) slope and bias correction (SBC), (**E**) calibration transfer method based on canonical correlation analysis (CCACT), (**F**) generalized least squares (GLSW), (**G**) multiplicative signal correction (MSC), (**H**) Recalibration and (**I**) partial least squares regression subspace based calibration transfer (PLSCT).

**Figure 3:**
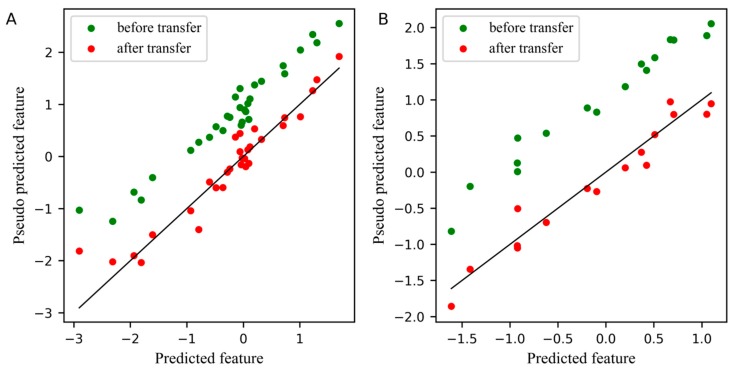
Plot for the differences between the feature before and after transfer in the partial least squares regression (PLS) subspace. (**A**) The differences of the first pseudo predicted feature of slave instrument standard set before and after transfer in PLS subspace, (**B**) The differences of the first pseudo predicted feature of slave instrument test set before and after transfer in PLS subspace.

In addition, the measured values of the moisture content of the corn dataset obtained from different models are compared with the predicted values when the number of the samples in the standard set is set to 30 are shown in Figure 2. In this case, the slope of the line was equal to 1. A point on the line indicates that the predicted value was equal to the measured value. As shown in Figure 2, PLSCT exhibited the smallest differences between the measured values and predicted values. This is attributed to the implementation of the feature transfer in the PLS subspace. The detailed description is shown in Figure 3.

For comparison, the differences between the feature before and after transfer in the PLS subspace, the relationship between the first pseudo predicted feature of the slave instrument and the first predicted feature of the master instrument is displayed in Figure 3. In these two plots, the blue dots represent the feature before transfer, and the red dots represent the feature after transfer. The closer the dots are to a straight line, the smaller the differences between the pseudo predicted feature of the slave instrument and the predicted feature of the master instrument. Figure 3A,B depicts the differences between features in the standard set and the test set, respectively. Obviously, after transfer, the differences between the first pseudo predicted feature of the slave instrument and the first predicted feature of the master instrument was significantly reduced, not only in the standard set, but also in the test set.

In order to evaluate the effect of the number of the samples in the standard set on different calibration methods, 5, 10, 15, 20, 25, and 30, standard samples were considered in the experiment. As can be seen from Table A1 in the Appendix A, the RMSEP of MSC was relatively large, and the predictability of CCACT and GLSW were better than that of PDS, SBC and MSC. From 5 samples to 30 samples, the RMSEP of PLSCT was smaller than the RMSEP of PDS, SBC, CCACT, GLSW and MSC. Moreover, the RMSEP of PLSCT had been gradually stabilized when the number of the samples in the standard set from 20 to 30. So, we conclude that PLSCT had significantly better predictive performance than other models.

To further compare PLSCT with other models, the RMSEP improvement and *p*-value by Wilcoxon signed rank test are listed in Table A2 in the Appendix A. The RMSEP improvement of PLSCT to PDS(3), PDS(5), PDS(7), SBC, CCACT, GLSW, MSC, Recalibration2 and Recalibration were as high as 35.00575%, 34.99841%, 34.98937%, 41.95097%, 37.18537%, 30.21822%, 85.7502%, 8.610493% and 2.26298%, respectively. The Wilcoxon signed rank test shows statistically significant differences between PLSCT and other models (include Recalibration) at the 95% confidence level. 

### 2.2. The Analysis of the Wheat Dataset

In Table 1, we can note that when no calibration transfer method was used, the difference between the RMSEP of directly using Calibration and the RMSEP of Recalibration was much smaller than the difference in corn dataset, in part because the difference between the two instruments in wheat dataset was relatively small.

**Figure 4:**
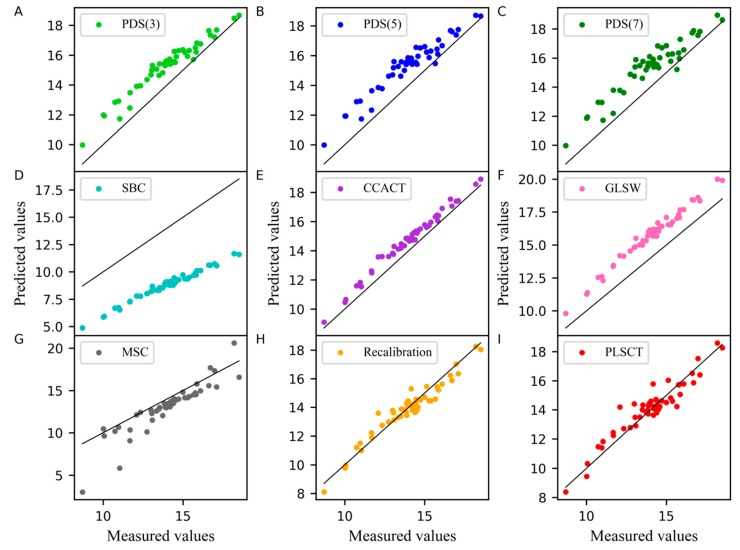
Measured values versus predicted values of protein content for wheat dataset as determined by (**A**) PDS(3), (**B**) PDS(5), (**C**) PDS(7), (**D**) SBC, (**E**) CCACT, (**F**) GLSW, (**G**) MSC, (**H**) Recalibration and (**I**) PLSCT.

Figure 4 displays the comparison of the measured values and the predicted values from different models. From these plots, it is worth noting that the differences between measured values and predicted values in PLSCT were only slightly larger than Recalibration and smaller than any other methods.

**Figure 5:**
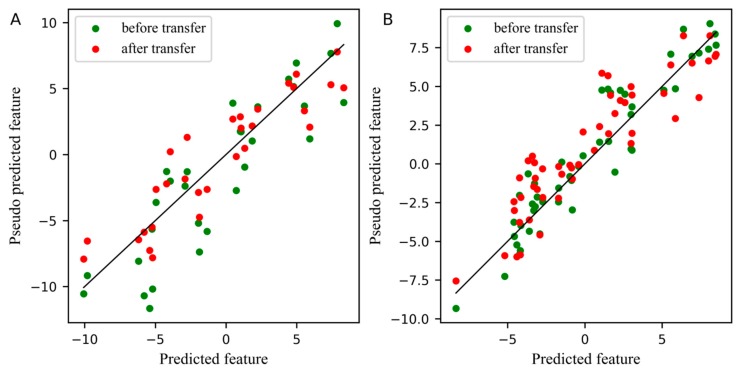
Plot for the differences between the feature before and after transfer in the PLS subspace. (**A**) The differences of the first pseudo predicted feature of slave instrument standard set before and after transfer in PLS subspace. (**B**) The differences of the first pseudo predicted feature of slave instrument test set before and after transfer in PLS subspace.

Since the spectra difference between the master instrument and the slave instrument was small in the wheat dataset, the effect of feature transfer was not obvious in the PLS subspace from Figure 5. However, the difference between the first pseudo predicted feature after transfer and the first predicted feature is still slightly smaller. The number of samples of the standard set in Figure 5A was 30.

The performances of the different methods on wheat samples are also shown in Appendix A Table A1. The Table A2 shows clearly that PLSCT has much lower prediction error than PDS, SBC, GLSW and MSC when the number of the samples in the standard set is 10, 25 and 30. When the number of the samples in the standard set was 30, the minimum RMSEP obtained by PLSCT was 0.6604. The RMSEP of Recalibration2 fluctuated greatly, probably because there were outliers in the standard set of the slave instrument. These outliers also affect the performance of the SBC as shown in Figure 4D.

The results by Wilcoxon signed rank test reveal that PLSCT is significantly different from PDS(3), PDS(5), PDS(7), SBC, CCACT, GLSW, MSC and Recalibration2 at 95% confidence level. The RMSEP improvement resulting from PLSCT compared with these models were 51.77389%, 54.35396%, 57.02112%, 87.45319%, 42.18862%, 61.34526%, 56.43832% and 69.98222%, respectively (shown in Appendix A Table A2).

### 2.3. The Analysis of the Pharmaceutical Tablet Dataset

As in the previous cases, the LVs and RMSEP of Calibration, Direct transfer and Recalibration are shown in Table 1. The RMSEP of Calibration is 3.123115, the RMSEP of direct transfer is 4.514284, the RMSEP of Recalibration was 3.31598.

**Figure 6:**
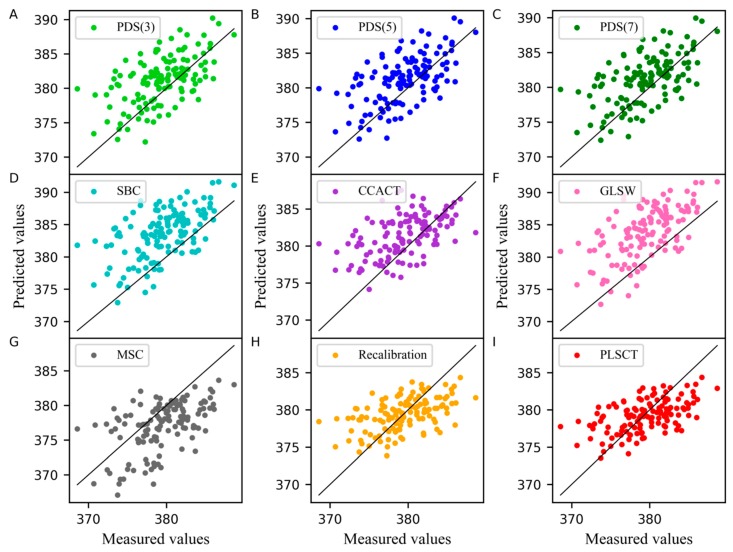
Measured values versus predicted values of pharmaceutical tablet dataset as determined by (**A**) PDS(3), (**B**) PDS(5), (**C**) PDS(7), (**D**) SBC, (**E**) CCACT, (**F**) GLSW, (**G**) MSC, (**H**) Recalibration and (**I**) PLSCT.

In the PLSCT model, the number of factors for constructing ${\tilde{T}}_{\mathrm{std}}^{s\_m}$ and ${\hat{T}}_{\mathrm{std}}^{m}$ was 4 when the number of the samples in the standard set was set to 25 and 30, as shown in Figure 1C,D. When the number of the samples in the standard set was set to 30, the comparison between the predicted values and measured values is shown in Figure 6. The results show that PLSCT has achieved the best performance.

**Figure 7:**
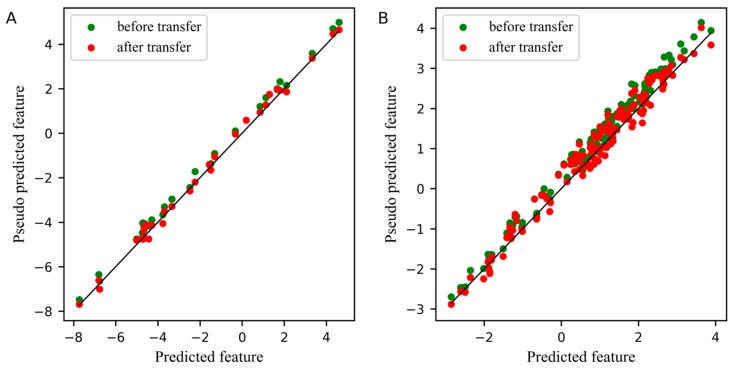
Plot for the differences between the feature before and after transfer in the PLS subspace. (**A**) The differences of the first pseudo predicted feature of slave instrument standard set before and after transfer in PLS subspace, (**B**) The differences of the first pseudo predicted feature of slave instrument test set before and after transfer in PLS subspace.

Figure 7 displays the comparison of the first pseudo predicted feature of the slave instrument standard set and test set before and after transfer in the PLS subspace, where the number of samples of the standard set in Figure 7A was 30. From the two plots in Figure 7, the first pseudo predicted feature after transfer was significantly closer to the predicted feature of the master instrument, whether in the standard set or in the test set of the slave instrument.

From Appendix A Table A1, as the number of the samples in the standard set increases, the performance of PLSCT gradually got better. The RMSEP of PLSCT gradually became stable when the number of samples in the standard set was 25 and 30, which were outperformed than PDS, SBC, CCACT, GLSW and MSC significantly. From the results in Table A2, when the number of the samples in the standard set was greater than 20, the RMSEP of PLSCT was already less than that of Recalibration.

Compared with other models, the RMSEP improvement of PLSCT over them can reach up 16.3743%, 15.12146%, 14.35178%, 40.04516%, 16.81376%, 41.83697%, 24.21448%, 23.82937% and 2.908651%, respectively. Furthermore, the differences between PLSCT and other models are all statistically significant at the 95% confidence level (shown in Appendix A Table A2).

## 3. Materials and Methods

### 3.1. Dataset Description

#### 3.1.1. Corn Dataset

**Figure 8:**
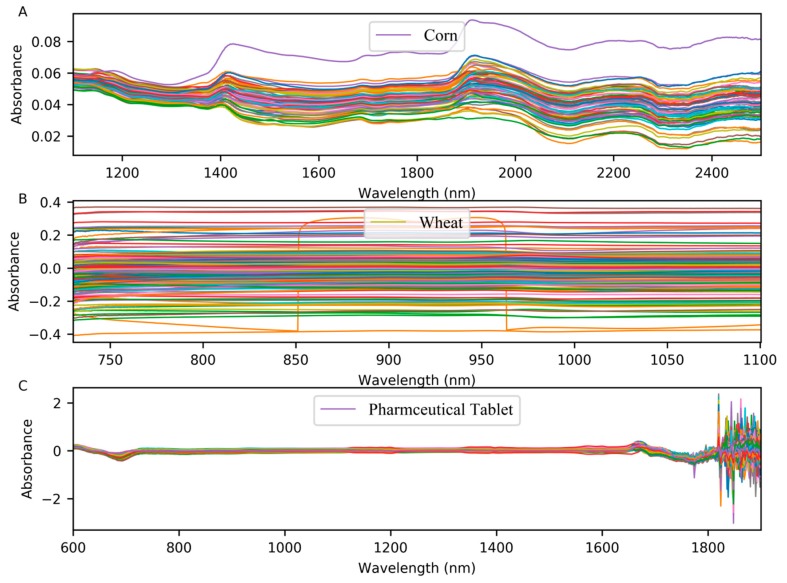
(**A**) The difference between the spectra of corn samples measured on M5 and MP5; (**B**) the difference between the spectra of wheat samples measured on A1 and A2; (**C**) the difference between the spectra of pharmaceutical tablet dataset.

The first dataset was corn dataset. We can conveniently access to obtain it at http://www.eigenvector.com/data/Corn/. The dataset is composed of 80 corn samples. Three near-infrared spectrometers were used to measure these samples, with wavelength range from 1100 nm to 2498 nm at 2 nm intervals (700 channels). The property of moisture, oil, protein and starch of corn is contained in the dataset. In this paper, the moisture content was chosen as the property of interest. We choose M5 as ‘master instrument’, MP5 as ‘slave instrument’. The difference between the spectra measured on M5 instrument and MP6 instrument can be observed in Figure 8A.

#### 3.1.2. Wheat Dataset

The second dataset was the wheat dataset, which consisted of 248 samples measured by three instruments of manufacturer A. This dataset was the shootout data of the International Diffuse Reflectance Conference (IDRC) in 2016. We can obtain it from http://www.idrc-chambersburg.org/content.aspx?page_id=22&club_id=409746&module_id=191116. The wavelength range of the manufacturer A was 730 nm–1100 nm and the interval was 0.5 nm. The dataset only provides the reference protein values. In this paper, we take the first instrument of manufacturer A as ‘master instrument’ and the second instrument as ‘slave instrument’. Figure 8B shows the difference between the spectra measured on the A1 and A2 instruments.

#### 3.1.3. Pharmaceutical Tablet Dataset

The third dataset came from the IDRC shootout 2002, which contains 655 pharmaceutical tablets measured on two spectrometers, with the range from 600 to 1898 nm, and the interval was 2 nm. We can obtain it from http://www.eigenvector.com/data/tablets/index.html. There are three reference values associated with this dataset, but we were only interested in weight content for each sample. The difference between the spectra in the pharmaceutical tablet dataset is shown in Figure 8C.

### 3.2. Dataset Division

We adopt the Kennard and Stone algorithm [28] to split the dataset. Firstly, the entire samples were split into the calibration set and the test set. The test set accounted for 20% of the total samples, and the remaining 80% was used as the calibration set. The corn dataset was divided into 64 samples for calibration set and 16 samples for the test set. The wheat dataset was divided into 198 samples for calibration set and 50 samples for the test set. For the pharmaceutical tablets dataset, we first integrated the three parts that have been divided, and then divided it into 524 samples for calibration sets and 131 samples for test sets. The standard samples were selected from the calibration set via the Kennard and Stone algorithm.

It must be noted that the Kennard and Stone algorithm was applied to the master spectra when splitting the calibration set and test set, while the Kennard and Stone algorithm was applied to the slave spectra when extracting the standard samples.

### 3.3. Determination of the Optimal Parameters

The number of latent variables used in the PLS model was selected by a 10-fold cross-validation. In order to avoid over-fitting caused by the inclusion of redundant latent variables, the optimal number of latent variables was achieved based on the statistical F-test [29] (*α* = 0.05).

The predicted feature from the standard set of slave instrument is a pseudo predicted feature ${\tilde{T}}_{\mathrm{std}}^{s\_m}$ constructed by the PLS model of the master instrument. Compared with the predicted feature ${\tilde{T}}_{\mathrm{std}}^{s}$ constructed by the PLS model of the slave instrument, the ${\tilde{T}}_{\mathrm{std}}^{s\_m}$ may contain some noise, which has a great influence on the solution of the transfer matrix ξ, further affecting the performance of the PLSCT model. In order to optimize the model, we used leave-one-out cross-validation to select the best number of factors in the standard set based on the minimum root mean square error of cross-validation (RMSECV) criterion. The response variable of the standard set used in cross-validation was the predicted value of the master instrument standard set obtained by the PLS model of the master instrument.

For the PDS method, its window sizes were set to 3, 5, and 7, respectively.

### 3.4. Model Performance Evaluation

In order to verify the prediction performance of different calibration models, we calculated the root mean square error of prediction (RMSEP). The calculation of RMSEP is as follows:

(1) $$\mathrm{RMSEP}=\sqrt{\frac{\sum_{i=1}^{n}{\left(y_{i}-{\hat{y}}_{i}\right)}^{2}}{n}}$$

where $y_{i}$ represents the measured value associated to the i-th test sample, ${\hat{y}}_{i}$ is its final predicted value, while n is the number of samples in the test set.

In order to compare the prediction performance difference between the proposed model and other models more directly, Equation (2) was used to calculate the RMSEP improvement of the PLSCT method compared with other methods:

(2) $$h=\left(1-\frac{{\mathrm{RMSEP}}_{\mathrm{PLSCT}}}{{\mathrm{RMSEP}}_{\mathrm{other}}}\right)\times 100\%$$

where ${\mathrm{RMSEP}}_{\mathrm{PLSCT}}$ represents the prediction error of the PLSCT method, ${\mathrm{RMSEP}}_{\mathrm{other}}$ represents the prediction error of other comparison methods.

In addition, by comparing prediction error of the different models, the Wilcoxon signed rank test at the 95% confidence level was utilized to point out whether there was a significant difference between PLSCT and other methods. In python, we used the wilcoxon function in the scipy package to directly calculate the *p*-value between the two prediction errors. If *p* > 0.05, there is no significant difference between the two methods. Otherwise, there is significant difference.

### 3.5. Calibration Transfer Method

#### 3.5.1. Notation

In this paper, we define the spectral matrix as X, n×p represents the size of the matrix, n represents the number of samples, p represents the number of variables, and $x_{i}$ represents the spectral variables corresponding to the i-th sample of the matrix. The response variables are defined as y and the predicted values are defined as $\hat{y}$. In order to distinguish the spectra collected on the two instruments, we added a superscript to the back of the matrix, such as defining the spectra from the master instrument as $X^{m}$, defining the spectra from the slave instrument as $X^{s}$, the predicted feature matrix of the master spectra obtained by the master instrument calibration model is ${\hat{T}}^{m}$, the pseudo predicted feature matrix of the slave spectra obtained by the master instrument calibration model is ${\tilde{T}}^{s\_m}$. At the same time, a subscript was added to the back of the matrix to distinguish different data sets. For instance, $X_{\mathrm{cal}}^{m}$, $X_{\mathrm{std}}^{m}$, and $X_{\mathrm{test}}^{m}$ represent the calibration set, standard set and test set of the master instrument, respectively. $X_{\mathrm{cal}}^{s}$, $X_{\mathrm{std}}^{s}$, and $X_{\mathrm{test}}^{s}$ represent the calibration set, standard set and test set of the slave instrument, respectively.

#### 3.5.2. Overview of PLS

PLS is a widely used multivariate calibration technique. PLS applies score vectors model the relationship between X and y. It projects X and y into a PLS subspace, a low-dimensional space defined by a small number of the score vectors. The mean-centered X and y are decomposed as follows:

(3) $$\left\{ \begin{array}{l} X=TP^{T}+E\\ y=Tq^{T}+F \end{array}\right.$$

where T is the score matrix, P and q represent loadings matrix for X and y, respectively. E and F are the matrices of residuals corresponding to X and y.

The matrix of regression coefficients is:

(4) $$\beta =W{\left(P^{T}W\right)}^{-1}q^{T}$$

where W is the weight matrix.

With the regression coefficient matrix β, we can have the predicted values:

(5) $$\hat{y}=X\beta$$

#### 3.5.3. Proposed PLSCT method

In the PLSCT, the PLS model was built on the calibration set of the master instrument to construct the PLS subspace, which is also the feature space constructed by the feature vectors of the spectra of the master instrument calibration set. The number of latent variables (LVs) in the PLS model is determined by cross-validation.

(6) $$\beta^{m}=W^{m}{\left({\left(P^{m}\right)}^{T}W^{m}\right)}^{-1}{\left(q^{m}\right)}^{T}$$

On the basis of this PLS model, the predicted feature matrix of standard set in the master instrument $X_{\mathrm{std}}^{m}$ can be calculated via it, that is, the spectra of the master instrument can be projected into the PLS subspace:

(7) $${\hat{T}}_{\mathrm{std}}^{m}=X_{\mathrm{std}}^{m}W^{m}{\left({\left(P^{m}\right)}^{T}W^{m}\right)}^{-1}$$

Similarly, the pseudo predicted feature matrix of standard set in the slave instrument $X_{\mathrm{std}}^{s}$ can be calculated via this PLS model as well as $X_{\mathrm{std}}^{m}$, in other words, the spectra of the slave instrument can be projected into this PLS subspace:

(8) $${\tilde{T}}_{\mathrm{std}}^{s\_m}=X_{\mathrm{std}}^{s}W^{m}{\left({\left(P^{m}\right)}^{T}W^{m}\right)}^{-1}$$

The two predicted feature matrices obtained are derived from the same PLS model of the master instrument, that is to say, all spectra are projected into the identical PLS subspace constructed by the master instrument. In the identical PLS subspace, there should be a linear relationship between the two feature matrices. So ${\tilde{T}}_{\mathrm{std}}^{s\_m}$ and ${\hat{T}}_{\mathrm{std}}^{m}$ can be built as:

(9) $${\tilde{T}}_{\mathrm{std}}^{s\_m}\xi ={\hat{T}}_{\mathrm{std}}^{m}$$

The linear relationship between the two feature matrices can be solved through the ordinary least squares method, by the following equation:

(10) $$\xi ={\left({\left({\tilde{T}}_{\mathrm{std}}^{s\_m}\right)}^{T}{\tilde{T}}_{\mathrm{std}}^{s\_m}\right)}^{-1}{\left({\tilde{T}}_{\mathrm{std}}^{s\_m}\right)}^{T}{\hat{T}}_{\mathrm{std}}^{m}$$

Once ξ is computed, for the test set from the slave instrument $X_{\mathrm{test}}^{s}$, applying Equation (11) to calculate the predicted values corresponding to the spectra:

(11) $${\hat{y}}_{\mathrm{test}}=X_{\mathrm{test}}^{s}W^{m}{\left({\left(P^{m}\right)}^{T}W^{m}\right)}^{-1}\xi {\left(q^{m}\right)}^{T}$$

## 4. Conclusions

In this paper, an ingenious calibration transfer method based on PLS subspace is proposed. PLSCT uses the same PLS model to project the spectra into the identical PLS subspace. In the identical subspace, a feature transfer model is constructed by narrowing the differences between the predicted feature of master instrument and the pseudo predicted feature of the slave instrument via an ordinary least squares method. Additional, PLSCT does not need the response variable corresponding to the standard set. As expected, experimental results on three real datasets show that compared with PDS, SBC, CCACT, GLSW, and MSC, the PLSCT model is more stable and can obtain more accurate prediction results. The reason why the PLSCT model can achieve such remarkable results is that while the spectra of the slave instrument are projected into this subspace, some noise effects such as scattering that are unrelated to the response variable will be removed from the spectra, and then the feature transfer in the identical PLS subspace can more accurately narrow the differences between the predicted feature of master instrument and the pseudo predicted feature of slave instrument.

## Figures and Tables

**Table 1:** Root mean square error of prediction (RMSEP) obtained by Calibration, Direct transfer, and Recalibration on three spectra datasets.

Instrument	Methods	LVs	RMSEP
Corn	Calibration ^1^	13	0.010156
	Direct transfer ^2^		1.41931
	Recalibration ^3^	5	0.208522
Wheat	Calibration ^1^	12	0.258014
	Direct transfer ^2^		0.85131
	Recalibration ^3^	8	0.530799
Pharmaceutical tablet	Calibration ^1^	7	3.123115
	Direct transfer ^2^		4.514284
	Recalibration ^3^	2	3.31598

^1^ Calibration: the calibration model of the calibration set of the master instrument; ^2^ Direct transfer: the calibration model of master instrument is used on the slave instrument without modification; ^3^ Recalibration: the calibration model of the calibration set of the slave instrument.

## Appendix A

**Table A1:** RMSEP for three datasets using different transfer methods.

	PDS			SBC	CCACT	GLSW	MSC	PLSCT	Recalibration2 ^2^	Recalibration ^3^
	W ^1^ = 3	W ^1^ = 5	W ^1^ = 7							
Corn dataset										
N = 5	0.4142	0.4336	0.4354	0.5370	0.2411	0.4056	1.4302	0.1991	0.3538	0.2085
N = 10	0.3753	0.3617	0.3729	0.4440	0.2545	0.3696	1.4302	0.1980	0.2237	
N = 15	0.3507	0.3495	0.3357	0.4307	0.3663	0.3535	1.4302	0.2127	0.2425	
N = 20	0.3440	0.3440	0.3440	0.3900	0.2841	0.3208	1.4302	0.2087	0.2379	
N = 25	0.3373	0.3372	0.3366	0.3720	0.3528	0.3106	1.4302	0.2082	0.2314	
N = 30	0.3136	0.3135	0.3135	0.3511	0.3245	0.2921	1.4302	0.2038	0.2230	
Wheat dataset										
N = 5	8.2434	9.1587	8.4226	14.3731	1.6248	4.0835	1.5160	1.8478	2.7176	0.5308
N = 10	8.5844	9.3534	10.8927	10.5310	1.2496	3.5824	1.5160	0.8588	2.2233	
N = 15	2.1373	2.8513	3.2012	8.7159	1.5315	2.9205	1.5160	1.8280	1.3985	
N = 20	1.9586	2.0927	2.2380	7.0482	0.9688	2.4743	1.5160	1.8263	0.4520	
N = 25	1.5656	1.6480	1.7445	6.1945	1.0437	1.9804	1.5160	0.6850	2.3661	
N = 30	1.3694	1.4468	1.5366	5.2635	0.7735	1.7085	1.5160	0.6604	2.2000	
Pharmaceutical tablet dataset										
N = 5	4.7971	4.2899	4.4594	5.9983	4.1302	6.5988	4.2482	3.3202	5.8027	3.3160
N = 10	4.1431	4.0098	4.0444	5.4720	4.1112	5.6721	4.2482	3.5821	5.5904	
N = 15	3.9698	3.8314	3.8347	5.7069	3.9357	6.2284	4.2482	3.3834	5.8043	
N = 20	3.9787	3.8789	3.9190	5.2838	3.8979	5.6511	4.2482	3.2794	5.0811	
N = 25	3.9263	3.8416	3.7789	5.2514	4.0549	5.4809	4.2482	3.2765	4.9428	
N = 30	3.8499	3.7931	3.7590	5.3699	3.8703	5.5354	4.2482	3.2195	4.2267	

^1^ w stands for window size of PDS method; ^2^ Recalibration2: the calibration model of the standard set of the slave instrument; ^3^ Recalibration: the calibration model of the calibration set of the slave instrument.

**Table A2:** RMSEP comparison of PLSCT and other methods, RMSEP improvement and *p*-values by the Wilcoxon signed rank test (α = 0.05) (the number of samples in the standard set is 30).

		PLSCT		
		Corn	Wheat	Pharmaceutical Tablet
PDS(3) ^1^	*h* (%) ^2^	35.00575	51.77389	16.3743
	*p* ^3^	**0.00717**	**3.17 ×** $10^{-9}$	**3.2 ×** $10^{-19}$
PDS(5) ^1^	*h* (%)	34.99841	54.35396	15.12146
	*p*	**0.00717**	**2.23 ×** $10^{-9}$	**1.78 ×** $10^{-19}$
PDS(7) ^1^	*h* (%)	34.98937	57.02112	14.35178
	*p*	**0.00717**	**2.23 ×** $10^{-9}$	**1.2 ×** $10^{-18}$
SBC	*h* (%)	41.95097	87.45319	40.04516
	*p*	**0.011286**	**7.56 ×** $10^{-10}$	**4.84 ×** $10^{-23}$
CCACT	*h* (%)	37.18537	42.18862	16.81376
	*p*	**0.004455**	**0.001161**	**4.37 ×** $10^{-21}$
GLSW	*h* (%)	30.21822	61.34526	41.83697
	*p*	**0.00717**	**8.53 ×** $10^{-10}$	**6.82 ×** $10^{-23}$
MSC	*h* (%)	85.7502	56.43832	24.21448
	*p*	**0.000531**	**1.57 ×** $10^{-6}$	**1.51 ×** $10^{-18}$
Recalibration2	*h* (%)	8.610493	69.98222	23.82937
	*p*	**0.017378**	**0.000231**	**3.05 ×** $10^{-23}$
Recalibration	*h* (%)	2.26298	−24.4164	2.908651
	*p*	0.876722	9.06 × $10^{-5}$	**0.000198**

^1^ The number in brackets stands for window size of PDS method; ^2^ *h*: the RMSEP improvement; ^3^ *p*: *p*-value by Wilcoxon signed rank test.
